# Inhibition of p38 MAPK decreases hyperglycemia-induced nephrin endocytosis and attenuates albuminuria

**DOI:** 10.1007/s00109-022-02184-5

**Published:** 2022-04-22

**Authors:** Magdalena Patrycja Woznowski, Sebastian Alexander Potthoff, Eva Königshausen, Raphael Haase, Henning Hoch, Catherine Meyer-Schwesinger, Thorsten Wiech, Johannes Stegbauer, Lars Christian Rump, Lorenz Sellin, Ivo Quack

**Affiliations:** 1 Department of Nephrology, Medical Faculty, Heinrich-Heine University, 40225 Düsseldorf, Germany; 2 Emergency Department, Klinikum Konstanz, 78464 Konstanz, Germany; 3 Institute of Pathology, Nephropathology Section, University Medical Center Hamburg-Eppendorf, 20246 Hamburg, Germany; 4 Institute of Cellular and Integrative Physiology, University Clinic Hamburg-Eppendorf, 20246 Hamburg, Germany

**Keywords:** Diabetes, Nephrin, Podocyte, Endocytosis, Albuminuria

## Abstract

**Abstract:**

Chronic hyperglycemia, as in diabetes mellitus, may cause glomerular damage with microalbuminuria as an early sign. Noteworthy, even acute hyperglycemia can increase glomerular permeability before structural damage of the glomerular filter can be detected. Despite intensive research, specific antiproteinuric therapy is not available so far. Thus, a deeper understanding of the molecular mechanisms of albuminuria is desirable. P38 MAPK signaling is involved in the development of hyperglycemia-induced albuminuria. However, the mechanism of increased p38 MAPK activity leading to increased permeability and albuminuria remained unclear. Recently, we demonstrated that acute hyperglycemia triggers endocytosis of nephrin, the key molecule of the slit diaphragm, and induces albuminuria. Here, we identify p38 MAPK as a pivotal regulator of hyperglycemia-induced nephrin endocytosis. Activated p38 MAPK phosphorylates the nephrin c-terminus at serine 1146, facilitating the interaction of PKCα with nephrin. PKCα phosphorylates nephrin at threonine residues 1120 and 1125, mediating the binding of β-arrestin2 to nephrin. β-arrestin2 triggers endocytosis of nephrin by coupling it to the endocytic machinery, leading to increased glomerular permeability. Pharmacological inhibition of p38 MAPK preserves nephrin surface expression and significantly attenuates albuminuria.

**Key messages:**

- Acute hyperglycemia triggers endocytosis of nephrin.
- Activated p38 MAPK phosphorylates the nephrin c-terminus at serine 1146, facilitating the interaction of PKCα with nephrin.
- PKCα phosphorylates nephrin at threonine residues 1120 and 1125, mediating the binding of β-arrestin2 to nephrin.
- β-arrestin2 triggers endocytosis of nephrin by coupling it to the endocytic machinery, leading to a leaky glomerular filter.
- Pharmacological inhibition of p38 MAPK preserves nephrin surface expression and significantly attenuates albuminuria under hyperglycemic conditions.

**Supplementary Information:**

The online version contains supplementary material available at 10.1007/s00109-022-02184-5.

## Introduction

Malfunction of the three-layered glomerular filter causes albuminuria which is an independent factor of morbidity and mortality and accelerates kidney disease progression [1]. Due to their exposed localization, podocytes encounter many pathologic stimuli. In particular, hyperglycemia can rapidly affect podocyte and slit diaphragm function, compromising the integrity of the glomerular filter [2].

Nephrin, a single transmembrane protein, functions as the backbone of the slit diaphragm and is therefore pivotal for its function. The deficiency of nephrin leads to a severe form of hereditary nephrotic syndrome [3]. Previous studies have suggested that nephrin is also critically involved in the pathogenesis of acquired proteinuric diseases. We recently demonstrated that acute hyperglycemia is sufficient to cause loss of nephrin by endocytosis and albuminuria. High glucose levels increase the binding of protein kinase C alpha (PKCα) to the intracellular part of nephrin. PKCα then phosphorylates nephrin threonine residues 1120 and 1125, creating a binding motif for β-arrestin2. The multifunctional adaptor β-arrestin2 couples nephrin to the endocytotic machinery and triggers the internalization of nephrin [4, 5]. These observations supported the hypothesis that a loss of nephrin from the slit diaphragm leads to increased permeability of the slit diaphragm and consequently to albuminuria. Our initial discovery of nephrin endocytosis has now evolved as an emerging focus on slit diaphragm biology [6–9]. There are many other kinase cascades, besides PKCα signaling, that are activated in a diabetic milieu. One member of the MAPK family, p38 mitogen-activated protein kinase (p38 MAPK), has recently been linked to diabetes and proteinuric kidney disease [10, 11].

Activation of p38 MAPK, which is triggered by phosphorylation of p38 MAPK residues Threonine 180 and Tyrosine 182, can be induced by many stimuli, including inflammatory cytokines, mechanical stretch, UV-radiation, osmotic shock, and high glucose [12]. Studies on human and experimental diabetes found activation of the p38 MAPK signaling cascade in glomeruli and tubules in the early and advanced stages of diabetes [13]. Closer analysis of the glomeruli revealed increased levels of phosphorylated p38 MAPK in mesangial cells, endothelial cells, and podocytes. There are a plethora of known phosphorylation targets of p38 MAPK, including nuclear transcription factors (i.e., ATF-2), other kinases (i.e., MK-2), or transmembrane receptors (i.e., EGFR) [14]. Nephrin is a receptor-like single transmembrane protein. Immunofluorescence studies of diabetic kidneys have shown a change in nephrin distribution from a linear to a coarse granular pattern. These findings resemble the pattern that can be found in the experimental nephrotic syndrome caused by adriamycin [15]. We recently demonstrated that this pattern change was at least partially due to the internalization of nephrin [16]. Thus, we were interested in whether endocytosis of nephrin might also occur in hyperglycemia-induced alteration of the slit diaphragm that predates histopathological changes as seen in diabetic kidney disease. Since treatment of animals with a p38 MAPK inhibitor preserved the linear pattern, p38 MAPK signaling might play a role in nephrin endocytosis. However, the molecular mechanism that links p38 MAPK to nephrin endocytosis and proteinuria remained unclear so far. In the present paper, we investigate the role of p38 MAPK in mediating hyperglycemia-induced albuminuria by controlling slit diaphragm integrity.

## Method and materials

### Study approval

This study was carried out in strict accordance with the recommendations in the Guide for the Care and Use of Laboratory Animals of the National Institutes of Health. All experiments were in accordance with the German/European law for animal protection and were approved by the local ethic committees.

### Hyperglycemia mouse model and treatment

Mice were obtained either from an in-house breed at a local animal care facility or from Janvier Labs, France. As an in vivo model, hyperglycemia was induced by intraperitoneal injection of streptozotocin as previously described [17, 18]. In brief, 6 to 8-week-old male C57Bl/6 mice were treated for 5 days. On day 1, mice were injected intraperitoneal either with 5 µL/g bodyweight of 0.9% NaCl as control or 5 µL/g bodyweight of streptozotocin (STZ) at a final concentration of 150 µg/g bodyweight. On days 2–4, controls were treated with 5 µL/g bodyweight of 0.9% NaCl. STZ mice were treated from day 2–4 daily with either 5 µL/g bodyweight of 0.9% NaCl or 5 µL/g bodyweight SB202190 hydrochloride, a pyridinyl imidazole-based p38 MAPK inhibitor, at a final concentration of 5 µg/g bodyweight via intraperitoneal injection. 12-h urine collection and blood sampling occurred on day 5, as well as euthanasia and organ removal. Only animals that developed hyperglycemia were included in the study.

### Cell culture

#### Immortalized murine podocytes

Immortalized murine podocytes were generously provided by Dr. Peter Mundel (Goldfinch Bio, Cambridge, MA). The podocytes were grown on type I collagen under permissive temperature (33 °C) in the presence of 10 units/mL IFN-γ in culture medium (RPMI + 10% FCS). To induce differentiation, the cells were maintained at 37 °C without IFN-γ for 10–14 days [19]. For experiments, the podocytes were exposed to normal glucose (5.5 mM) for 24 h and to high glucose (30 mM) for the time points described in the figure legends.

RPMI culture medium and FCS were obtained from Biochrom (Germany).

#### Human embryonic kidney 293 cells (HEK293T cells)

HEK293T cells were grown in 10 cm culture dishes in culture medium (DMEM/F-12 medium + 10% FCS) at 37 °C and 5% CO2. Confluent cells were carefully washed with PBS, trypsin added for 3–5 min at 37 °C, and then rinsed off with culture medium. Cell stock was split into new 10 cm culture dishes and grown in a culture medium for around two days until confluence was reached. For experiments, the cells were exposed to normal glucose (5.5 mM) or to high glucose (30 mM) without FCS for 24 h prior to the time points described in the figure legends.

DMEM/F-12 culture medium and FCS were obtained from Biochrom (Germany).

#### Calcium phosphate transfection of HEK293T

HEK293T cells were transiently transfected with plasmid DNA by using the calcium phosphate method, as described before [20]. Briefly, 10 µg DNA was added to 500 µL of 0.25 M CaCl_2_, mixed and added dropwise to 500 µL of 2× HEPES buffer, vortexed gently, and incubated for 5 min. The cells were incubated with this solution for 6–8 h and harvested after 18 h.

2× HEPES buffer: 280 mM NaCl, 10 mM KCl, 1.5 mM Na_2_HPO_4_–H_2_O, 12 mM dextrose, 50 mM HEPES.

### Reagents

All reagents were purchased from Sigma-Aldrich unless stated otherwise. The p38 MAPK inhibitor was obtained from Calbiochem/Merck Millipore. Specifically, the following antibodies and reagents were used: p-p38 (catalog #4511, or #9211, cell signaling Massachusetts, USA), p38 (catalog #8690, cell signaling), nephrin (catalog #GP-N2, Progen, Heidelberg, Germany), β-actin (catalog #A5316, Sigma-Aldrich, St. Louis, Missouri, USA), streptavidin (Thermo Scientific, Waltham, Massachusetts, US), PKCα (catalog #sc-208, Santa Cruz, Dallas, Texas, USA), p-Nephrin (S1146, generated by Eurogentec), and p-Nephrin (Thr1120/Thr1125, generated by Eurogentec).

### Plasmids

C-terminal FLAG-tagged β-arrestin2 was a generous gift from Dr. Robert Lefkowitz (Duke University, Durham, NC). Human nephrin cDNA, as described previously [21], was kindly provided by Dr. Gerd Walz (University of Freiburg, Freiburg, Germany). The S1146A nephrin mutant (forward primer 5′ GGG ACT TCG CCC CCC AGC TGC CCC CGA CGC AGG 3′, reverse primer 5′ GGC AGC TGG GGG GCG AAG TCC CTC AGG GAG CGG 3′) was generated by site-directed mutagenesis. In brief, PCR was performed with the primers mentioned. The PCR product was purified by phenol extraction and further treated with restriction enzyme *Dpn I* and thereafter transformed into competent bacterial cells [22]. Nephrin S1146A was cloned MluI/NotI into pCDM8 vector.

Membrane-bound fusion proteins of the C-terminal cytoplasmic domains of nephrin were generated using a pCDM8 cassette that contained the leader sequence of CD5 fused to the CH2 and CH3 domains of human IgG1 followed by the transmembrane region of CD7 [23]. Dr. Jae-Won Soh (Inha University, Incheon, Korea) provided the expression plasmid of PKCα, and Dr. Lindsay Hinck (Western Washington University, San Francisco, CA) provided the expression constructs of protein interacting with c kinase-1 (PICK1), which have been described previously [24]. C-terminal FLAG-tagged β-arrestin2 was a generous gift from Dr. Robert Lefkowitz (Duke University, Durham, NC).

### Coimmunoprecipitation

Coimmunoprecipitations were performed as described previously [23]. In brief, HEK293T cells were transiently transfected by the calcium phosphate method. After incubation, the cells were washed twice and lysed in 1% Triton X-100 lysis buffer. After centrifugation (15,000 × *g* for 15 min at 4 °C), cell lysates containing equal amounts of total protein were incubated for 1 h at 4 °C with the appropriate antibody, followed by incubation with 30 μL of protein G-sepharose for 3 h. Sepharose was washed extensively with lysis buffer, and the bound proteins were resolved by 10% SDS-PAGE and visualized by Western blotting.

### Recombinant proteins

GST-nephrin fusion proteins were generated by cloning the nephrin gene or gene fragments into the pGEX-4 T-1 vector. The constructs were transformed into *Escherichia coli* BL21 (Novagen, Merck, Nottingham, UK), and expression of the recombinant proteins was induced by isopropyl-β-d-thiogalactoside. The GST-nephrin fusion proteins were subsequently purified from bacterial extracts by affinity chromatography using glutathione-sepharose (ÄKTAprime; GE Healthcare, Freiburg, Germany) according to the manufacturer's protocol. Recombinant GST-tagged β-arrestin2 was purchased from Abnova (Heidelberg, Germany), and ATF2 was obtained from Sigma-Aldrich/Merck.

### Kinase assay

Recombinant p38 MAPK was purchased from Sigma-Aldrich/Merck. The kinase reaction was performed in reaction buffer (20 mM HEPES, pH 7.4, 0.03% Triton X-100, 100 μg/mL phosphatidylserine, 10 μg/mL diacylglycerol, 10 mM MgCl_2_, 0.1 mM CaCl_2_, 50 μM ATP, and 5 μCi of [γ-32P]ATP) for 30 min at 30 °C. The reaction was stopped by adding 4× sample buffer and heating for 5 min at 90 °C. Following separation on SDS–polyacrylamide gels, the gels were fixed in 50% methanol and 10% acetic acid, dried and exposed to x-ray film.

### Analysis of albuminuria

Twelve-hour urine collection was performed in single metabolic cages on day 5. Albuminuria was assessed using a 10% polyacrylamide gel. Urinary albumin and creatinine excretion were measured using standard laboratory protocols, and albuminuria was normalized based on creatinine excretion.

### In vivo and in vitro biotin labeling and immunoprecipitation (IP)

First, kidneys were perfused via the abdominal aorta with 5 mL ice-cold PBS (rate 2 mL/min) supplemented with 1 mM MgCl_2_ and 0.1 mM CaCl_2_ (PBSCM). Subsequently, perfusion was repeated with 5 mL PBSCM (rate 2 mL/min) supplemented with 0.5 mg/mL EZ-Link™ sulfo-NHS-LC-biotin (Thermo Scientific, Rockford, USA) for surface protein labeling. Afterward, unspecific biotin-binding was quenched with 5 mL PBSCM (rate 2 mL/min) supplemented with 100 mM glycine. Finally, kidneys were perfused with 5 mL PBSCM (rate 2 mL/min) containing 16 × 10^6^ dynabeads/mL (Invitrogen, Oslo, Norway). After perfusion, kidneys were immediately minced and digested for 40 min at 37 °C with 1.5 mg/mL Collagenase A (Roche, Mannheim, Germany). Cell suspensions were filtered through 100 µm cell strainers (Greiner Bio-One, Frickenhausen, Germany), separated by centrifugation (5.000 × *g* for 5 min at 4 °C), and washed with PBSCM using a Dynamag magnet. When purity of > 95% glomeruli was achieved, glomeruli were collected by centrifugation (6.800 × *g* for 5 min at 4 °C). Samples were immediately homogenized using a TissueRuptor (Qiagen, Hombrechtikon, Switzerland) and were lysed for 30 min on ice. Insoluble cellular material was removed by centrifugation (15,000 × *g* for 30 min at 4 °C). Protein concentrations from the supernatant were measured using a BCA Protein assay kit (Thermo Scientific, Rockford, USA) and were subsequently adjusted to ensure equal total protein content.

In vitro, HEK293T cells were transfected with human nephrin cDNA or its mutant nephrin S1146A in 5.5 mM glucose containing DMEM/F-12 plus FCS as described above. Cell culture medium was changed 24 h prior to cell harvesting to either 5.5 mM Glucose or 30 mM glucose without FCS. The cells were harvested in ice-cold PBSCM (see above). Plasma membrane proteins were labeled with EZ-Link™ sulfo-NHS-LC-biotin (0.5 mg/mL) for 30 min at 4 °C. Unbound biotin was quenched twice with ice-cold PBSCM containing 100 mM glycine.

Cells were lysed in 1% Triton X-100 lysis buffer.

#### IP analysis:

Samples were incubated with anti-nephrin antibodies overnight at 4 °C (in vivo) or 1-h (in vitro), followed by a 3-h incubation (in vivo) or 1-h incubation (in vitro) with protein A-sepharose (GE LifeSciences, Freiburg, Germany) or with streptavidin agarose beads directly (Pierce, Thermo Fisher Scientific, Waltham, USA). Due to N-linked glycosylation, nephrin appears as a double band [25]. The immunoprecipitates were extensively washed with CHAPS buffer (in vivo) or Triton 1% lysis buffer (in vitro). Bound proteins were resolved using 2× Laemmli sample buffer, separated on 10% polyacrylamide gel, and electroblotted onto nitrocellulose membranes. The blots were blocked in 5% bovine serum albumin (BSA) in TBST before incubation with primary antibodies overnight at 4 °C. After washing with TBST for 30 min, the blots were incubated for 60 min with HRP-coupled secondary antibodies, and excessive antibodies were removed by washing with TBST for 30 min. ECL SuperSignal (Thermo Scientific, Rockford, U.S.A.) was used for chemiluminescence visualization (FluorChem FC2 Imager; Alpha Innotec, USA), and the densitometric analysis was performed (AlphaView SA; Cell Biosciences Inc., version 3.3.1, Alpha Innotec, USA).

### Immunofluorescence

For immunofluorescent staining, 2 µm paraffin sections from kidneys (day 5) were deparaffinized, and antigen retrieval was performed by boiling at 98 °C in 0.05% citraconic acid anhydride (Sigma, St. Louis, USA), pH 7.4 for 40 min. Unspecific binding was blocked in 5% horse serum with 0.05% Triton X-100 for 30 min at room temperature (RT). Primary antibody incubations (guinea-pig α-nephrin (IF 1:200, Acris, Rockville, USA) and rabbit α-EEA1 (IF 1:400, Santa Cruz, Santa Cruz, USA) were performed in blocking buffer overnight at 4 °C. Binding was visualized by incubation with AF488-α-guinea pig or Cy3-α-rabbit coupled secondary antibodies (all affinity-purified donkey antibodies Jackson ImmunoResearch, West Grove, USA) diluted 1:400 in blocking buffer for 30 min at RT. Sections were evaluated with a Zeiss LSM 510 meta microscope using the LSM software (all Zeiss, Jena, Germany).

### Quantification and statistical analysis

Statistical analysis was done using unpaired *t*-test with Welch’s correction.

## Results

### Hyperglycemia induces phosphorylation (activation) of p38 MAPK in glomeruli and podocytes

**Fig. 1:**
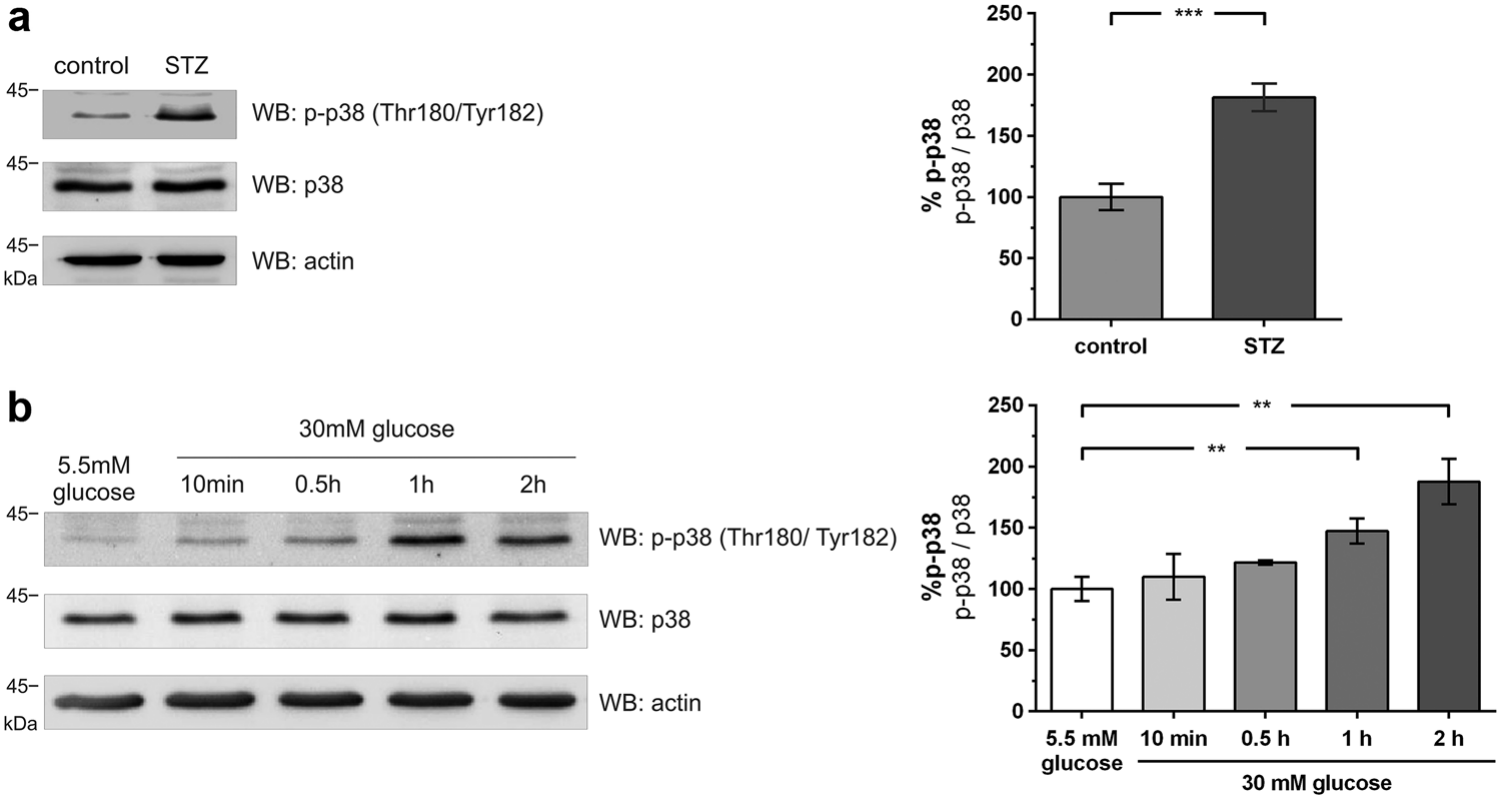
Hyperglycemia induces phosphorylation of p38 MAPK in glomeruli and podocytes. **a** Western blot analysis of phospho-p38 (WB: p-p38) and total p38 (WB: p38) expression in glomerular lysates of normoglycemic (control) and hyperglycemic (STZ) mice (day 5). β-actin (WB: actin) served as a loading control. The results of five independent experiments were quantified by densitometry and graphed as phospho-p38 (p-p38) to total p38 (p38) ratio – the control group was normalized to 100%. Control (*n* = 5) vs. STZ (*n* = 6): 100.0 ± 10.8 vs. 181.5 ± 11.2% (***p < 0.001); **b** Western blot analysis of phospho-p38 and total p38 expression in immortalized murine podocytes under normoglycemic (5.5 mM) and high (30 mM) glucose conditions at the given time points. The results of five independent experiments were quantified by densitometry graphed as phospho-p38 (p-p38) to total p38 (p38) ratio – normoglycemic (5.5 mM) group was normalized to 100%. A total of 5.5 mM glucose (*n* = 6) vs. 30 mM glucose 1 h (*n* = 6)/30 mM glucose 2 h (*n* = 6): 100.0 ± 9.9 vs. 147.3 ± 10.3% / 187.6 ± 18.5% (**p < 0.01 vs. control)

In a first step, we examined p38 MAPK expression and activity in murine glomeruli and podocytes exposed to pathologic glucose levels. Hyperglycemia was induced in C57/Bl6 mice with streptozotocin (STZ). Hyperglycemic animals showed a significant increase of phosphorylated p38 MAPK in the glomerular lysates compared to their normoglycemic littermates (Fig. 1a).

Since the glomerular lysates comprised multiple different cell types that may have generated a phospho signal, we further analyzed immortalized podocytes exposed to a high glucose milieu (30 mM). Compared to normoglycemic glucose (5.5 mM), an increase in p38 MAPK phosphorylation starting 10 min after a medium change to high glucose occurred, reaching its peak at 2 h (Fig. 1b).

### Inhibition of p38 MAPK prevents hyperglycemia-induced albuminuria

**Fig. 2:**
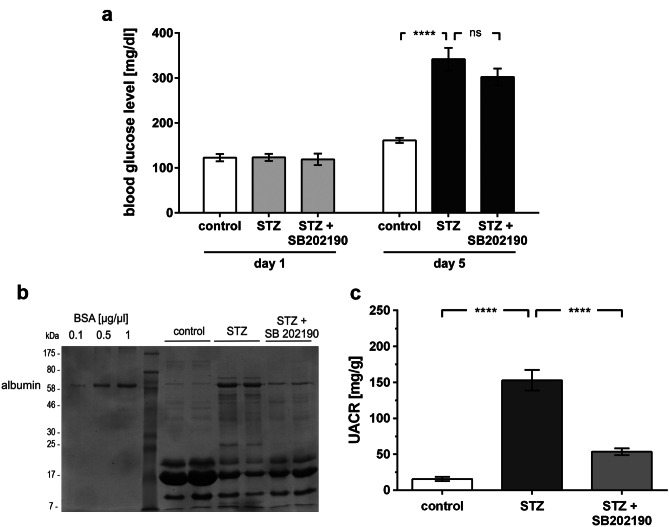
Inhibition of p38 MAPK does not alter development of hyperglycemia but prevents hyperglycemia-induced albuminuria. **a** Blood glucose levels of normoglycemic (control), hyperglycemic (STZ), and hyperglycemic mice treated with p38 MAPK inhibitor (STZ + SB202190) at day 1 and day 5. Glucose levels day 5: control (*n* = 11) vs. STZ (*n* = 14): 161.0 ± 5.6 vs. 341.6 ± 25.3 mg/dL (****p < 0.0001); STZ (*n* = 14) vs. STZ + SB202190 (*n* = 16): 341.6 ± 25.3 vs. 302.1 ± 18.8 mg/dl (*p* = ns). **b** Molecular mass markers are indicated in kilodalton (kDa), SDS-PAGE/Coomassie gel staining of urine (day 5) in normoglycemic (control), hyperglycemic (STZ), and hyperglycemic mice treated with p38 MAPK inhibitor (STZ + SB202190). BSA at 1, 5, and 10 µg/µL served both as a control and standard. **c** Quantitative analysis of the urinary albumin to creatinine ratio (UACR) (day 5) in normoglycemic (control), hyperglycemic (STZ), and hyperglycemic mice treated with p38 MAPK inhibitor (STZ + SB202190). UACR day 5: control (*n* = 18) vs. STZ (*n* = 15) 15.6 ± 3.0 vs. 152.9 ± 14.3 mg/g (****p < 0.0001); STZ (*n* = 15) vs. STZ + SB202190 (*n* = 8): 152.9 ± 14.3 vs. 53.5 ± 4.7 mg/g (****p < 0.0001)

To determine whether p38 MAPK is a mediator of hyperglycemia-induced albuminuria, we used the pyridinyl imidazole inhibitor SB202190 to block p38 MAPK kinase activity. SB202190 competes with ATP binding and is highly selective without effects on ERK or JNK below a concentration of 100 µM [26, 27]. Albuminuria was quantified in hyperglycemic C57/Bl6 mice on day 5. The treatment group was given SB202190. Blood glucose levels in treated and untreated animals showed no significant differences (Fig. 2a).

Compared to normoglycemic littermates, the hyperglycemic animals developed significant albuminuria. In hyperglycemic animals, the inhibition of p38 MAPK activity by SB202190 attenuated albuminuria significantly (Fig. 2b and c).

### p38 MAPK interacts with nephrin and phosphorylates nephrin at serine 1146

**Fig. 3:**
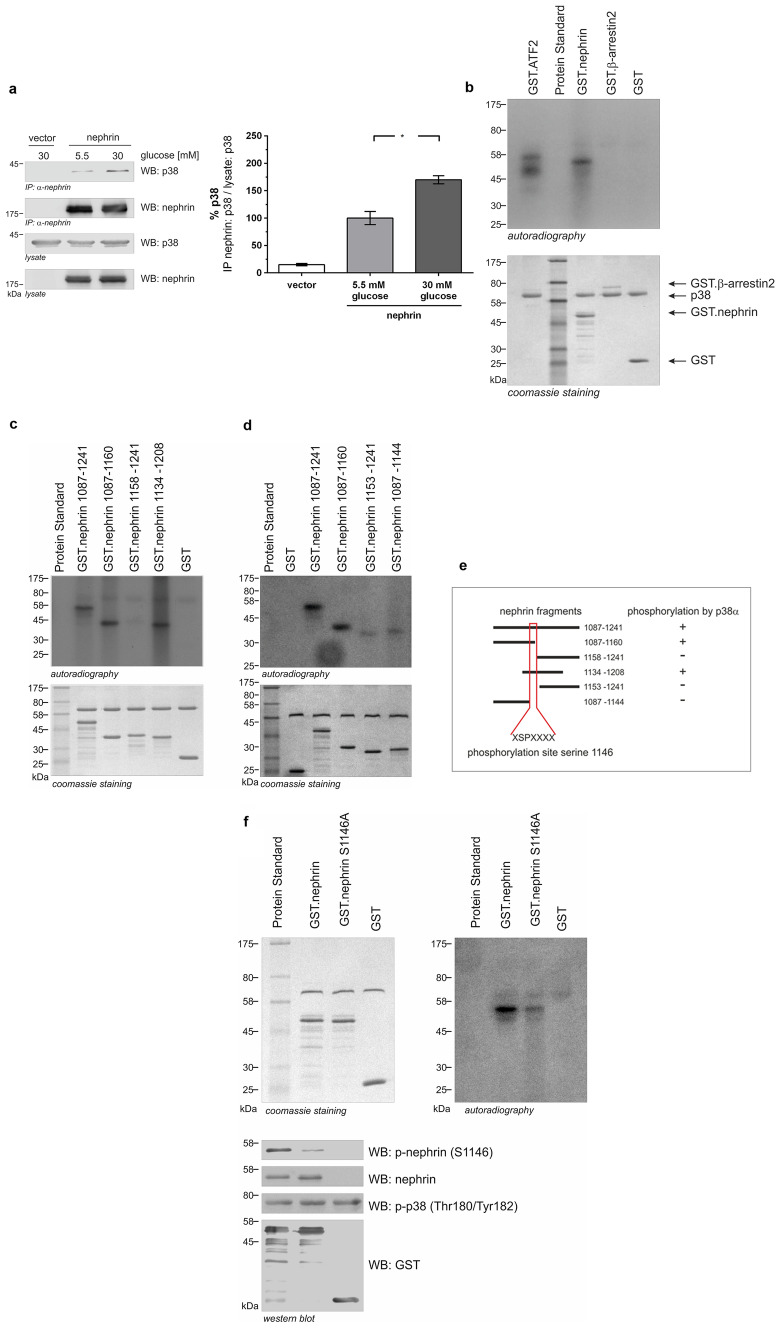
P38 MAPK interacts with nephrin and phosphorylates nephrin (molecular mass markers are indicated in kilodalton (kDa)). **a** Coimmunoprecipitation. HEK293T cells were transiently transfected with either vector or nephrin. Cells were either kept under normal (5.5 mM) or high (30 mM) glucose for 24 h. Nephrin was immobilized, and interaction with p38 MAPK was determined by p38-antibody staining. Compared to normal glucose conditions (5.5 mM), high glucose (30 mM) showed a significant increase in nephrin-p38 interaction (interaction was normalized to 100% for normal glucose conditions (5.5 mM)) ratio of *IP nephrin: p38 / lysate: p38* [%]: 5.5 mM vs. 30 mM (*n* = 3): 100.0 ± 11.9 vs. 169.9 ± 7.3% (*p* < 0.05). **b** In vitro phosphorylation assay. Aliquots of recombinant wild-type nephrin cytoplasmic domain (aa 1087–1241) and controls (GST.ATF2, GST.β-arrestin2, and GST) were expressed in *E. coli*. Subsequently, recombinant p38 MAPK and [γ-32P] were added. GST.ATF2 was used as the positive control. Phosphorylation was visualized by autoradiography. Coomassie staining of an SDS-PAGE gel showed equal protein input. **c**, **d** In vitro phosphorylation assay of different truncated nephrin cytoplasmic domains. Aliquots of recombinant wild-type nephrin cytoplasmic domain (amino acids (aa) 1087–1241) and different truncated nephrin cytoplasmic domains were expressed in *E. coli*. Following, recombinant p38 MAPK and [γ-32P] were added. Phosphorylation was visualized by autoradiography. Coomassie staining of an SDS-PAGE gel showed equal protein input. **e** Graphical summary of truncated proteins and phosphorylation status. **f** In vitro phosphorylation assay of nephrin cytoplasmic domain and mutated nephrin cytoplasmic domain S1146A. Aliquots of recombinant wild-type nephrin cytoplasmic domain (GST.nephrin; aa 1087–1241) and mutated nephrin cytoplasmic domain (GST.nephrinS1146A) and GST were expressed in *E. coli*. Subsequently, recombinant p38 MAPK and [γ-32P] were added. Phosphorylation was visualized by autoradiography. Coomassie staining of an SDS-PAGE gel showed equal protein input. Western blot analysis of phosphorylation of nephrin serine 1146 and p38 MAPK with phospho-specific antibodies. Staining of total nephrin and GST served as loading controls

In HEK293T, we discovered an interaction between p38 MAPK and nephrin. Increasing the glucose level to 30 mM led to an increase in this interaction (Fig. 3a). In view of our previous findings, it was our hypothesis that the c-terminus of nephrin might also be a phosphorylation target of p38 MAPK. To test this hypothesis, we performed in vitro kinase assays with recombinant nephrin and p38α MAPK. P38α MAPK induced robust phosphorylation of the known target ATF-2 as well as the intracellular domain of nephrin. β-arrestin2, serving as control, was not phosphorylated (Fig. 3b).

Next, different truncated proteins of the nephrin c-terminus were generated to delineate the phosphorylation motif. The truncated proteins comprising the amino acids (aa) 1087–1241, aa 1087–1160, and aa 1134–1208 showed a phospho-signal, while the protein fragment aa 1158–1241 did not (Fig. 3c). These results suggest that the amino acids 1134–1157 comprise the target motif for p38 MAPK phosphorylation. Two more truncated proteins were designed (aa 1153–1241 and aa 1087–1144), which narrowed the region of interest to amino acids 1145–1152 (Fig. 3d).

From these remaining residues, serine 1146, which is part of a classical xxPSxx sequence, was the most promising candidate (Fig. 3e).

Thus, we generated a non-phosphorylatable mutant and changed serine on position 1146 to alanine (S1146A). In comparison to wild-type nephrin, phosphorylation of the mutant S1146A was markedly decreased, indicating serine 1146 as a specific p38 MAPK phosphorylation target in the nephrin c-terminus (Fig. 3f).

### Decreased phosphorylation of serine 1146 attenuates binding of PKCα to nephrin c-terminus

**Fig. 4:**
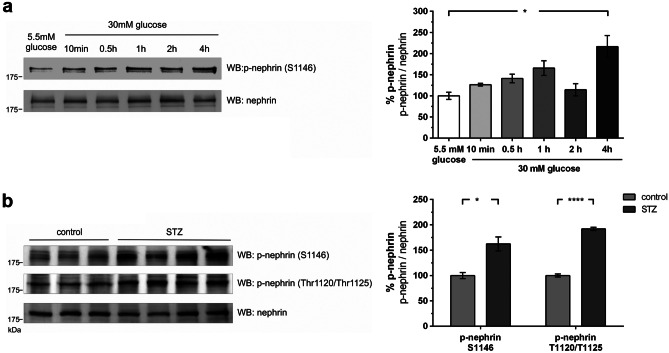
Phosphorylation of nephrin serine 1146 in murine podocytes and nephrin serine 1146 and threonine 1120/1125 in diabetic mice (molecular mass markers are indicated in kilodalton (kDa)). **a** Western blot analysis of phosphorylation of nephrin serine 1146 in immortalized murine podocytes under low (5.5 mM) and high glucose (30 mM) conditions at given time points (*n* = 4 each). The results were quantified by densitometry and graphed as phospho-nephrin (p-nephrin) to total nephrin (nephrin) ratio–normoglycemic (5.5 mM) group was normalized to 100%. A total of 5.5 mM glucose vs. 30 mM glucose 4 h: 100.0 ± 8.4 vs. 216.4 ± 26.4% (*p < 0.05). **b** Western blot analysis of phosphorylation of nephrin serine 1146 and threonine 1120/1125 in nondiabetic (control) and diabetic (STZ) mice (day 5)–control was normalized to 100%: S1146 control (*n* = 3) vs. STZ (*n* = 4): 100.0 ± 6.0 vs. 162.2 ± 13.8% (*p < 0.05); T1120/1125 control (*n* = 3) vs. STZ (*n* = 4): 100.0 ± 2.9 vs. 191.9 ± 3.2% (****p < 0.0001)

Having identified serine 1146 as a target of p38 MAPK, we investigated whether hyperglycemia leads to a change in phosphorylation of this serine residue in vitro and in vivo. For this, a phospho-specific antibody for the phospho-serine 1146 motif was generated. Experiments in immortalized murine podocytes showed a parallel increase in phosphorylation of p38 MAPK and serine 1146 under high glucose conditions (Fig. 4a).

These results were also confirmed in vivo. Phosphorylation of serine 1146 in glomeruli obtained from the STZ-treated mice was increased (Fig. 4b). In a previous study, we identified PKCα as a mediator of albuminuria. The binding of PKCα to nephrin is increased under hyperglycemic conditions. The binding of PKCα to nephrin results in the phosphorylation of the threonine residues 1120 and 1125 (Thr1120/Thr1125) of nephrin, facilitating the binding of the adaptor β-arrestin2, which initiates endocytosis of nephrin [5]. Thus, we hypothesized whether p38 MAPK works further upstream as a modulator of PKCα signaling. Analysis of the threonine 1120/1125 motif in glomeruli of hyperglycemic mice showed an increase in phosphorylation compared to normoglycemic controls (Fig. 4b).

**Fig. 5:**
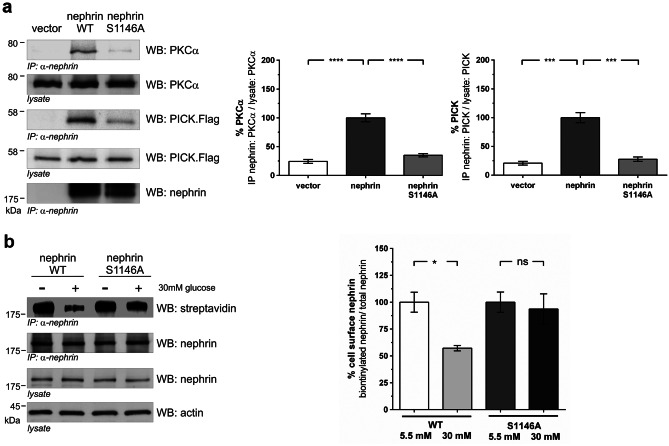
Interaction of PKCα with wild-type nephrin or mutated nephrin S1146A and biotinylation assay. **a** Western blot analysis of coimmunoprecipitation: HEK293T cells overexpressing untagged PKCα (PKCα), flag-tagged PICK1, and wild-type nephrin or mutated nephrin S1146A. Nephrin was immobilized, and the level of interaction was determined by staining of PKCα and PICK1. Staining of lysates of PCKα, PICK1, and nephrin served as loading controls. The results were quantified by densitometry and graphed as either the ratio of the PICK1 or PKCα coimmunoprecipitation signal intensity (IP nephrin: PICK1/lysate PICK1 or IP nephrin: PKCα/ lysate PKCα) to the lysate signal intensity (ratio lysate: PICK1 or lysate PKCα) – nephrin was normalized to 100%. %PKCα: nephrin (*n* = 6) vs. nephrin S1146A (*n* = 6): 100.0 ± 6.9 vs. 35.0 ± 2.9% (****p < 0.0001). %PICK: nephrin (*n* = 5) vs. nephrin S1146A (*n* = 5): 100.0 ± 8.8 vs. 27.7 ± 3.9% (***p < 0.001). **b** Biotinylation assay in HEK293T under normal (5.5 mM) and high (30 mM) glucose conditions. Immunoprecipitation of nephrin and the nephrin mutant S1146A (A). The biotinylated fraction of nephrin and its mutant was analyzed (WB: streptavidin). Staining of nephrin in the lysate and immunoprecipitation (WB nephrin) was performed. Actin (WB: actin) in the lysate served as loading controls. The results were quantified by densitometry and graphed as the ratio of biotinylated signal intensity to total nephrin signal intensity – 5.5 mM glucose group was normalized to 100%. A total of 5.5 mM glucose nephrin WT (*n* = 5) vs. 30 mM glucose nephrin WT (*n* = 5): 100.0 ± 9.3 vs. 57.2 ± 2.5% (*p < 0.05). A total of 5.5 mM glucose nephrin S1146A (*n* = 5) vs. 30 mM glucose nephrin S1146A (*n* = 5): 100.0 ± 9.4 vs. 93.6 ± 14.2% (*p* = ns)

Since we showed that hyperglycemia strengthened the binding of PKCα to nephrin, we investigated whether the interaction might be regulated by the phosphorylation status of serine 1146. Coimmunoprecipitation experiments in HEK293T cells showed that the interaction of PKCα and its adaptor PICK1 with the nephrin c-terminus was markedly impaired in the S1146A mutant compared to wild-type nephrin (Fig. 5a).

In HEK293T cells, increasing the glucose level from 5.5 mM to 30 mM caused a loss of surface nephrin shown in a biotinylation assay. This loss of surface nephrin was prevented in the nephrin S1146A mutant, indicating that nephrin phosphorylation at S1146 is crucial for nephrin endocytosis under hyperglycemic conditions (Fig. 5b).

### Inhibition of p38 MAPK decreases β-arrestin2 mediated nephrin endocytosis

**Fig. 6:**
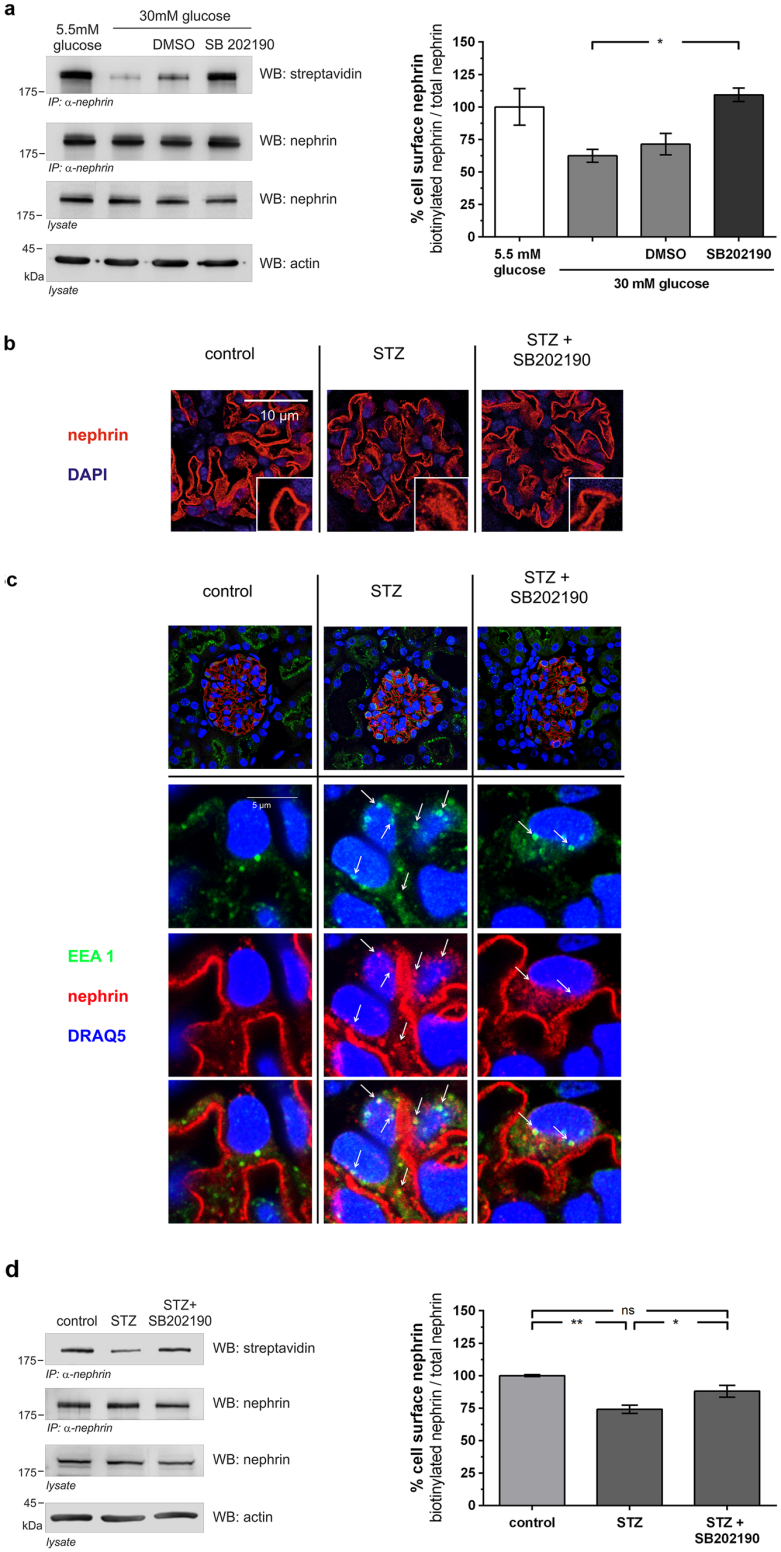
Inhibition of p38 MAPK decreases β-arrestin2 mediated nephrin endocytosis in vitro and in vivo. **a** Biotinylation assay in HEK293T under normal (5.5 mM) and high (30 mM) glucose conditions. Immunoprecipitation of nephrin was performed, and the biotinylated fraction of nephrin was analyzed (WB: streptavidin). Staining of nephrin in the lysate and immunoprecipitation (WB nephrin) and actin (WB: actin) in the lysate served as loading controls. The results were quantified by densitometry and graphed as the ratio of biotinylated signal intensity to total nephrin signal intensity – 5.5 mM glucose group was normalized to 100%. A total of 30 mM glucose (*n* = 3) vs. 30 mM glucose + SB202190 (*n* = 3): 62.4 ± 5.0 vs. 109.3 ± 5.2% (*p < 0.05). A total of 5.5 mM glucose (*n* = 3) vs. 30 mM glucose + SB202190 (*n* = 3):100.0 ± 14.1 vs. 109.3 ± 5.2% (*p* = ns). Data represent means ± SEM. Statistical analysis: unpaired t-test with Welch’s correction. **b** Representative immunofluorescence staining of murine kidney sections of normoglycemic (control), hyperglycemic (STZ), and hyperglycemic mice treated with p38 MAPK inhibitor (STZ + SB202190) (day 5, *n* = 3). Staining was performed with an anti-nephrin antibody (red) and nuclear DNA with DAPI (blue). **c** Representative immunofluorescence images of colocalization of nephrin with early endosomal antigen (EEA1) in immunofluorescence staining of murine kidney sections of healthy mice (control), untreated hyperglycemic mice (STZ), and hyperglycemic mice treated with p38 MAPK inhibitor (STZ + SB202190) (day 5, *n* = 3). Staining was performed with an anti-nephrin antibody (red), an anti-EEA1-antibody (green), and nuclear DNA with DRAQ5 (blue). White arrows indicate colocalization of nephrin with EEA1-positive vesicles. **d** Inhibition of p38 MAPK decreases β-arrestin2 mediated nephrin endocytosis in diabetic mice in a biotinylation assay of murine glomerular lysates of non-diabetic (control), diabetic (STZ), and diabetic mice treated with p38 MAPK inhibitor (STZ + SB202190) (day 5). Immunoprecipitation of nephrin was performed, and the biotinylated fraction of nephrin was analyzed (WB: streptavidin). Staining of nephrin in the lysate and immunoprecipitation (WB: nephrin) and actin (WB: actin) in the lysate served as loading controls. The results were quantified by densitometry and graphed as the ratio of biotinylated signal intensity to total nephrin signal intensity – the control group was normalized to 100%. Control (*n* = 4) vs. STZ (*n* = 4): 100.0 ± 0.7 vs. 74.2 ± 3.2% (**p < 0.01). STZ (*n* = 4) vs. STZ + SB202190 (*n* = 4): 74.2 ± 3.2 vs. 88.0 ± 4.5% (*p < 0.05). Control (*n* = 4) vs. STZ + SB202190 (*n* = 4): 100.0 ± 0.7 vs. 88.0 ± 4.5% (*p* = NS). Data represent means ± SEM. Statistical analysis: unpaired *t*-test with Welch’s correction

Taken together, our results suggested that p38 MAPK-mediated phosphorylation of nephrin S1146 regulates PKCα binding to nephrin. Thus, we finally wanted to confirm that p38 MAPK signaling regulates nephrin endocytosis. In HEK293T cells, increasing the glucose level from 5.5 mM to 30 mM caused a loss of surface nephrin. In contrast, inhibition of p38 MAPK by SB202190 treatment of cells preserved nephrin surface expression (Fig. 6a).

Representative immunofluorescence images were generated from mouse kidney samples of all groups to illustrate nephrin endocytosis. In normoglycemic mice, immunofluorescence of nephrin showed a linear pattern. In hyperglycemic mice, a change to a punctate pattern occurred, suggesting endocytosis of nephrin. Inhibition of p38 MAPK activity ameliorated the change in nephrin localization, and the linear staining pattern was mostly preserved in SB202190-treated animals (Fig. 6b). High-resolution images showed an enhanced colocalization of nephrin with early endosomal antigen 1 (EEA-1) in hyperglycemic animals, suggesting increased endocytosis of the nephrin molecules. In mice treated with SB202190, less colocalization was observed (Fig. 6c).

To quantify the amount of endocytosis, we took advantage of our biotin-based in vivo endocytosis assay [16]. Western blot analysis with streptavidin revealed the surface fraction of nephrin. Hyperglycemia caused a loss of approximately 26 ± 3% of nephrin from the slit diaphragm. Treatment with SB202190 decreased nephrin endocytosis. Approximately 88 ± 4% of nephrin remained on the cell surface (Fig. 6d).

**Fig. 7:**
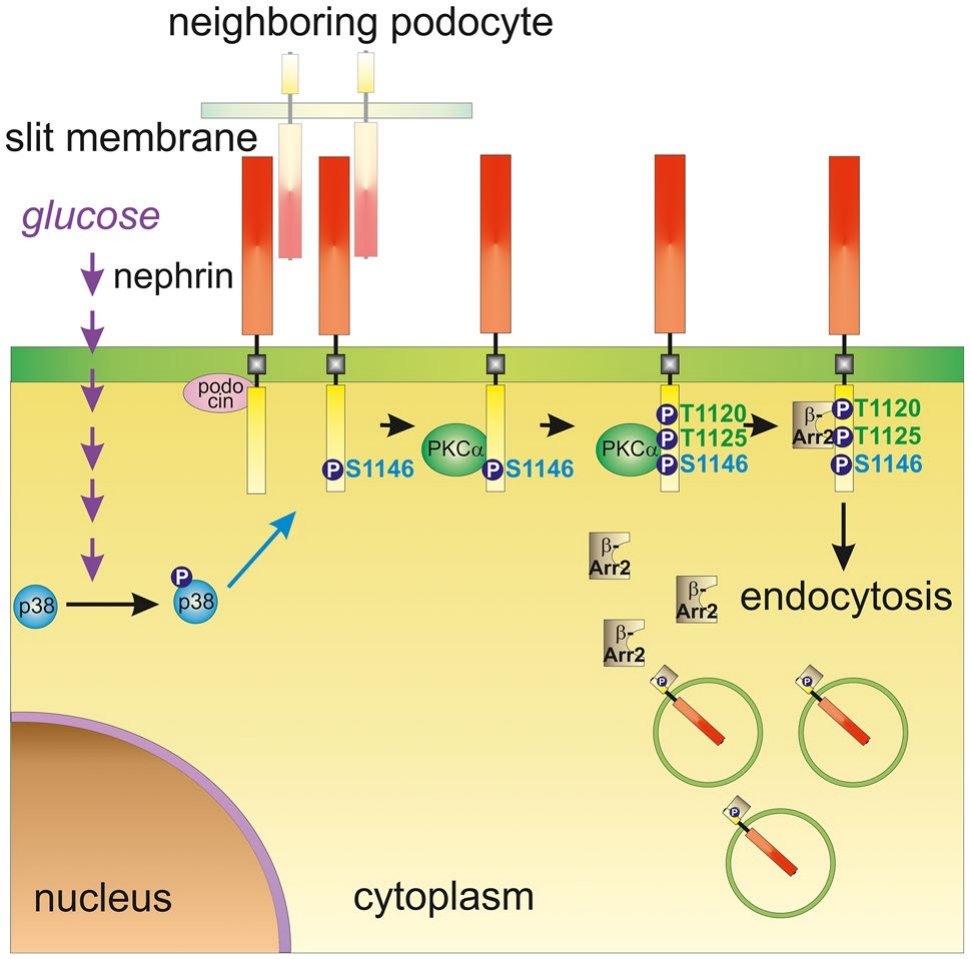
Molecular mechanism of hyperglycemia-induced nephrin endocytosis. High glucose levels activate p38 MAPK. p38 MAPK then phosphorylates nephrin at S1146, facilitating the interaction of PKCα with nephrin. PKCα phosphorylates nephrin at Thr1120/Thr1125, creating a β-arrestin2 biding site. β-arrestin2 couples nephrin to the endocytic machinery and triggers its internalization

Taken together, the present study suggests p38 MAPK as an important regulator of hyperglycemia-induced nephrin endocytosis and albuminuria (Fig. 7).

## Discussion

Hyperglycemia is able to activate p38 MAPK in a variety of renal cell types like podocytes, mesangial cells, and proximal tubular cells [28–30]. Activation of p38 MAPK under hyperglycemic conditions can promote apoptosis and inflammation [31, 32]. Modulation of the p38 MAPK pathway ameliorates hyperglycemia-induced kidney and renal cell injury [33–35].

In the presented study, we have identified p38 MAPK as an important regulator of hyperglycemia-induced nephrin endocytosis in vitro and in vivo. In vivo, the importance of the p38 MAPK signaling cascade for the integrity of the slit diaphragm was shown by inhibition of activated p38 MAPK in hyperglycemia, thus attenuating nephrin endocytosis and consecutively albuminuria. As we have previously demonstrated, hyperglycemia leads to endocytosis of nephrin via activation of PKCα and consecutive binding of β-arrestin2 to nephrin [5]. The presented study allows us to extend our model and show that p38 MAPK regulates PKCα signaling in the podocyte with a direct effect on nephrin endocytosis and albuminuria. Hyperglycemia activates p38 MAPK, which phosphorylates serine 1146 in the nephrin c-terminus. Phosphorylated serine 1146 facilitates PKCα binding to nephrin, consecutively increasing phosphorylation of the β-arrestin2 acceptor site in the nephrin c-terminus. In vitro, pharmacological inhibition of activated p38 MAPK preserves nephrin cell surface expression.

In line with these findings, inhibition of activated p38 MAPK in vivo preserves slit diaphragm integrity and attenuates albuminuria. Our initial observation was that hyperglycemia-induced robust phosphorylation of p38 MAPK in murine podocytes. Other groups have also observed significantly greater numbers of phospho-p38 positive podocytes in kidneys of diabetic mice [10]. Notably, Dai et al. found that glucose concentrations between 5.5 and 50 mM induced a linear increase in p38 MAPK phosphorylation [36]. Since p38 MAPK is able to regulate endocytosis of transmembrane proteins, such as FGFR1 and EGFR, by direct phosphorylation, we investigated whether the nephrin c-terminus was a target of p38 MAPK [37, 38]. Indeed, our kinase assays showed that the intracytoplasmic part of nephrin was phosphorylated by p38 MAPK. The signaling of p38 MAPK is normally associated with the phosphorylation of its substrates at serine-proline or threonine-proline motifs [14, 37, 38]. Intriguingly, the intracellular part of nephrin consisting of 154 amino acids harbors a serine-proline motif (aa 1144–1149: FDSPQL). In consecutive experiments with truncated nephrin proteins and mutants, we could determine nephrin serine 1146 as a phosphorylation target of p38 MAPK. A custom phospho-specific antibody revealed phosphorylation of nephrin serine 1146 under high glucose conditions in vitro and in vivo. In hyperglycemic mice that received the p38 inhibitor SB202190, phosphorylation of serine 1146 was attenuated. Furthermore, less phosphorylation of threonine 1120/1125, the phosphorylation motif of PKCα in the nephrin c-terminus, was detectable. In vitro, when serine 1146 was mutated to alanine (S1146A), the binding of PKCα to nephrin was significantly attenuated. In the in vitro biotinylation experiments, cell surface abundance of the nephrin mutant S1146A did not decrease significantly at high glucose (30 mM), indicating phosphorylation at S1146 to be crucial for nephrin endocytosis under hyperglycemic conditions.

These results show, for the first time, a specific role for serine phosphorylation in nephrin signaling. To quantify nephrin endocytosis in mice, we used our in vivo biotinylation assay as described previously [16]. Hyperglycemic mice showed significant albuminuria. In these mice, 25.83 +/− 3.25% of nephrin molecules were lost from the slit diaphragm. Complementary immunofluorescence studies of the murine kidneys revealed a change of the linear distribution of nephrin to a punctate pattern. Colocalization experiments showed that nephrin was translocated from the podocyte surface to the early endosome, suggesting endocytosis of nephrin. Inhibition of activated p38 MAPK preserved expression of nephrin at the slit diaphragm and attenuated albuminuria significantly. However, albuminuria was not attenuated completely, indicating that the p38 MAPK pathway is an important regulator but not solely responsible for the mediation of hyperglycemia-induced albuminuria.

In this study, the in vivo hyperglycemic model is induced with STZ in mice. In these animals, we observed a renal phenotype with increased albuminuria within 5 days. Induction of hyperglycemia using the STZ diabetes model is well established, and it is used in different intervention models [18, 39]. Palm et al. examined the STZ-diabetes model in rodents to differentiate between toxic effects of STZ and the specific effects of hyperglycemia. They showed that a STZ-based diabetes model induces proteinuria. In order to distinguish between alteration of the glomerular filtration barrier and tubular damage, they specifically addressed albuminuria as an important marker of increased permeability of the glomerular filtration barrier. Palm et al. demonstrated that albuminuria, but not tubular proteinuria, could be prevented by glycemic control in STZ-treated rats [40]. In the cited study, the onset of albuminuria in STZ-treated rats without glycemic control was detected from week 1 and increased over time. Glycemic control in STZ-treated rats abolished any albuminuria in this model. The early onset of albuminuria at week 1 predated any significant histopathological changes [40]. This indicates that disturbance of glomerular filtration with an increase in albuminuria occurs early in hyperglycemia and is primarily dependent on hyperglycemia rather than a direct effect of STZ. Therefore, in the presented study, we opted for a short time model of STZ-induced hyperglycemia to avoid histopathological alterations seen in a long-term diabetes model. Indeed, STZ-treated mice did not show any histopathological changes (light and electron microscopy – Fig. S1). Furthermore, the in vitro experiments in our study show a similar activation of the p38 MAPK signaling cascade under hyperglycemic conditions suggesting a STZ-independent mechanism. The work of Axelsson et al. provides additional evidence, indicating that hyperglycemia is sufficient to alter the selectivity of glomerular filtration. In nondiabetic rats, infusion of glucose caused a reversible increase in glomerular permeability, appearing within 20 min. These alterations were observed independently of the concomitant hyperosmolarity [2].

The significant effect of p38 MAPK inhibition in hyperglycemia-induced albuminuria raises expectations that inhibition of p38 MAPK might advance therapy of proteinuric diseases in general. Unfortunately, to date, clinical studies with p38 MAPK inhibitors in other inflammatory diseases did not achieve long-lasting effects. Interestingly, the antiproteinuric effect of p38 MAPK inhibition has also been described in other established models of nephrotic syndrome, including puromycin aminonucleoside and adriamycin nephropathy [11]. In this study, a similar characteristic change to a punctate pattern of nephrin expression was described as seen in our study, but the underlying molecular pathomechanism was not further elucidated. Taking advantage of our biotinylation assay, we recently demonstrated that this pattern change in adriamycin nephropathy and nephrotoxic nephritis is caused, at least in part, by nephrin endocytosis [16]. Besides our finding, identifying p38 MAPK as an important regulator of slit diaphragm integrity in hyperglycemia, it is intriguing to speculate whether p38 MAPK might also regulate nephrin endocytosis under other pathologic conditions.

## Supplementary Information

Below is the link to the electronic supplementary material.

Supplementary file1 (DOCX 634 KB)

## Data Availability

All data analyses during this study are included in this published article, and all data generated during the study are available from the corresponding author on request.
