# Supplementary material for: Inhibition of p38 MAPK decreases hyperglycemia-induced nephrin endocytosis and attenuates albuminuria
Source: J Mol Med (Berl). 2022 Apr 22;100(5):781–95. doi: 10.1007/s00109-022-02184-5 (PMC9110524; doi:10.1007/s00109-022-02184-5)
Supplement: Supplementary file 1 — Supplementary file1 (DOCX 634 KB) [file 109_2022_2184_MOESM1_ESM.docx]

JMME-D-20-00586

Inhibition of p38 MAPK Decreases Hyperglycemia-Induced Nephrin Endocytosis and Attenuates Albuminuria

Magdalena Patrycja Woznowski*,**,1, Sebastian Alexander Potthoff**,1, Eva Königshausen1, Raphael Haase1, Henning Hoch1, Catherine Meyer-Schwesinger4, Thorsten Wiech3, Johannes Stegbauer1, Lars Christian Rump1, Lorenz Sellin1, and Ivo Quack2

1 Department of Nephrology, Medical Faculty, Heinrich-Heine University, 40225 Düsseldorf, Germany

2 Emergency Department, Klinikum Konstanz, 78464 Konstanz, Germany

3 Institute of Pathology, Nephropathology Section, University Medical Center Hamburg-Eppendorf, 20246 Hamburg, Germany

4 Institute of Cellular and Integrative Physiology, University Clinic Hamburg-Eppendorf, 20246 Hamburg, Germany

**Supplement**

Kidney samples were assessed by a renal pathologist. PAS staining and electron microscopy were performed to evaluate glomerular- and tubule-structure and the glomerular filtration barrier (Fig. S1).

According to the histological assessment by the renal pathologist, neither PAS staining nor electron microscopy from each group showed any differences regarding the glomerulus, the glomerular filtration barrier or the tubules and interstitium.

**Fig. S1**

***
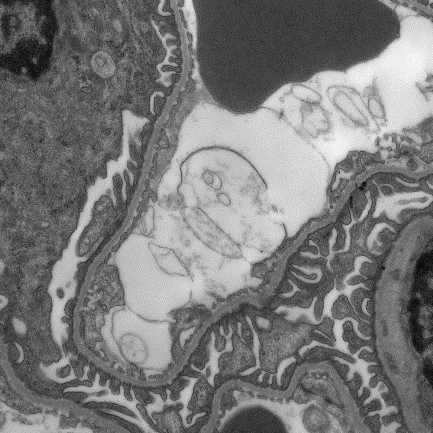

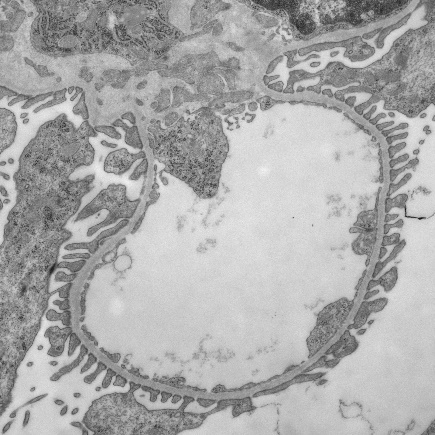

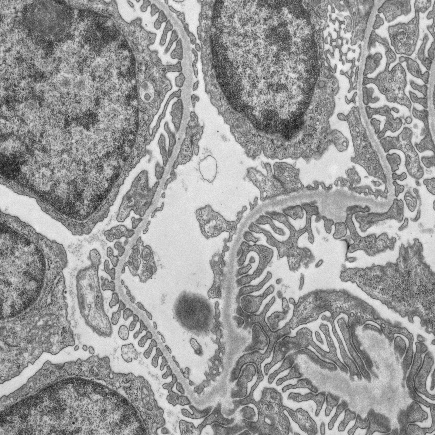
NaCl STZ STZ+SB202190***

***NaCl STZ STZ+SB202190***


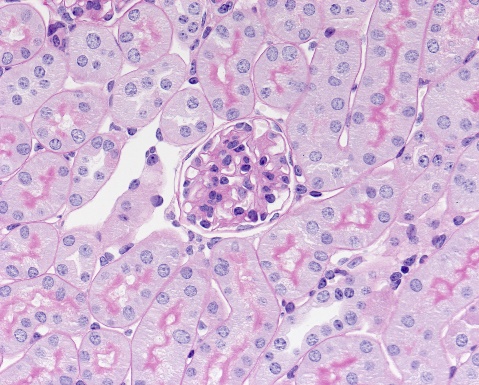

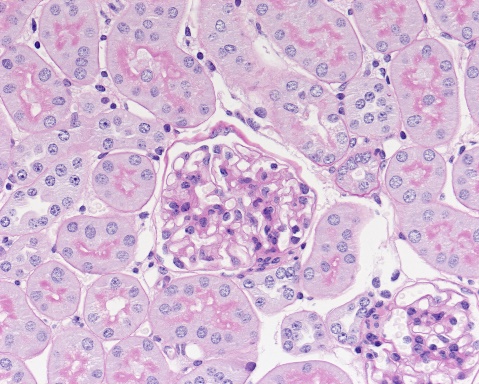

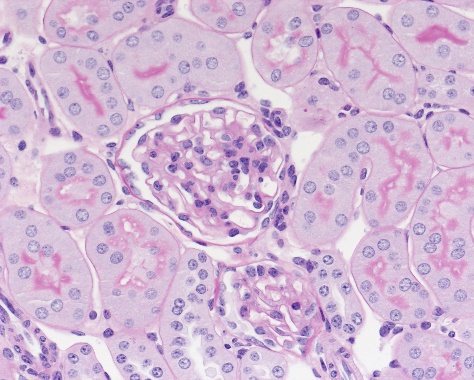


Fig. S1: Representative glomerular EM images and PAS staining from control, STZ and STZ+ SB 202190 treated mice.
